# A deep dive into selected work sectors during the COVID-19 pandemic and the “living with COVID” phase: understanding similarities and differences in practice, perceptions, and preparedness

**DOI:** 10.1093/annweh/wxad053

**Published:** 2023-09-23

**Authors:** Anna Coleman, Rebecca Canham, Katie Clabon, Paniz Hosseini, Sheena Johnson, Martie van Tongeren

**Affiliations:** Centre for Occupational and Environmental Health, School of Health Sciences, University of Manchester, Ellen Wilkinson Building, Oxford Road, Manchester M13 9PL, United Kingdom; Manchester Academic Health Science Centre, Manchester University, Nelson Street, Manchester M13 9NQ, United Kingdom; Behavioural Insights Team, Institute of Occupational Medicine, Research Avenue North, Riccarton, Edinburgh EH14 4AP, United Kingdom; Behavioural Insights Team, Institute of Occupational Medicine, Research Avenue North, Riccarton, Edinburgh EH14 4AP, United Kingdom; Department of Public Health, Environments and Society, London School of Hygiene and Tropical Medicine, Keppel Street, London, United Kingdom; People, Management and Organisations Division, Alliance Manchester Business School, University of Manchester, Booth Street West, Manchester M15 6PB, United Kingdom; Thomas Ashton Institute for Risk and Regulatory Research, University of Manchester and the Health and Safety Executive, Rm 6A.001, Core 1 Engineering Building A, The University of Manchester Booth Street East, Manchester M13 9PL, United Kingdom; Centre for Occupational and Environmental Health, School of Health Sciences, University of Manchester, Ellen Wilkinson Building, Oxford Road, Manchester M13 9PL, United Kingdom; Manchester Academic Health Science Centre, Manchester University, Nelson Street, Manchester M13 9NQ, United Kingdom; Thomas Ashton Institute for Risk and Regulatory Research, University of Manchester and the Health and Safety Executive, Rm 6A.001, Core 1 Engineering Building A, The University of Manchester Booth Street East, Manchester M13 9PL, United Kingdom

**Keywords:** COVID-19, industry response, prevention and control, protective practices, SARS-CoV-2, worker behaviours, workplace transmission, workplace transmission risk

## Abstract

**Objectives:**

When it comes to controlling workplace transmission of SARS-CoV-2, the virus that causes COVID-19, different workplaces and industrial sectors face different challenges, both in terms of likely transmission routes and which control measures can be practically, economically, and effectively implemented. This article considers a large body of research in the United Kingdom across different work sectors and time points during the COVID-19 pandemic to better understand mitigation measures, challenges to mitigating the risk of SARS-COV-2 transmission, knowledge gaps, and barriers and enablers to control viral transmission.

**Methods:**

Data is drawn from 2 phases of research. Phase 1 gathered data from an interactive workshop (April 2022) where PROTECT researchers working across 8 work sectors shared knowledge and expertise from research conducted between 2020 and 2022. Phase 2 revisited 6 of these sectors to explore participants’ views on the “living with COVID” phase of the pandemic (February–October 2022) through qualitative interviews.

**Results:**

Our findings emphasise the importance of considering the characteristics of each work sector (and their sub-sectors), relative to the physical workplace and workforce, the ways organisations operate, and how they interact with the public. Study findings show that participant’s views and organisational practices changed quickly and significantly over the course of the pandemic. Most participants initially perceived that the majority of risk mitigations would remain in place for the foreseeable future. However, following the change in Government Guidance towards “living with COVID”, most mitigation measures were quickly removed and it had become necessary for sectors/organisations to restore normal operations, thereby treating the COVID-19 virus like any other illness, while remaining prepared for future health emergencies that may arise.

**Conclusion:**

We suggest that national policy makers and organisational leaders remain mindful of the lessons learned and knowledge gained at all levels (national, regional, local, organisational, and individual) during the COVID-19 pandemic. We make recommendations in support of recovery as sectors/organisations continue “living with COVID” and other respiratory diseases; balanced with longer term planning for the next public health crisis.

**What’s Important About This Paper?**

Different workplaces and industrial sectors faced different challenges in controlling the transmission of SARS-CoV-2 during the COVID-19 pandemic. This study collated findings from 8 industrial sectors (between 2020 and 2022) and revisited 6 of these during the “living with COVID” pandemic phase (Autumn 2022) to better understand risks and responses to COVID-19 at the organisational and sectoral level. Understanding similarities and differences in practice, perceptions, and preparedness longitudinally across sectors is imperative to help continue short-term recovery and inform preparations for future health emergencies.

## Introduction

Between October 2020 and March 2023, the UK government funded the Partnership for Research in Occupational, Transport, and Environmental COVID-19 Transmission National Core Study (PROTECT NCS, Thomas Ashton Institute). This wide-ranging research programme aimed to improve understanding of risk of SARS-CoV-2 (the virus that causes COVID-19) transmission in workplaces and other environments. PROTECT consisted of 6 themes that use a complementary variety of research methods and scientific disciplines to address research questions from different perspectives (e.g. microbiology, building science, behavioural science, mathematical modelling, qualitative studies, etc.). Through PROTECT, a number of sector-specific studies (see Table 1) explored the implementation of risk mitigation methods, perceived risk of SARS-CoV-2 infection, and lived experiences of COVID-19 in different industrial sectors in the United Kingdom. These studies encompassed consultation with diverse stakeholders including, but not limited to, organisational leaders, industry experts (e.g. federations/associations, academics, policy makers), front-line workers, and where appropriate, members of the public. Study findings contributed to the development of (sector-specific) recommendations to support policy and decision-making and could support the development of more effective responses to infectious disease outbreaks in the future. Selection of the sectors was based on existing research and networks within the research team (e.g. construction, logistic deliveries, education), sectors identified as priorities early in the pandemic (e.g. public transport, care homes, and food and drink processing), and opportunities that arose during the pandemic for specific studies (energy production and close contact retail). Certain sectors were not included as the PROTECT study was focussing predominantly on sectors which were not already covered comprehensively by other research groups in the United Kingdom (e.g. health care).

**Table 1.** Sectors and representatives.

Sector	Reference to related sector-specific research	Knowledge share workshop (Phase 1)	Semi-structured interviews (Phase 2)	
		Participants (researchers)	Type of job role	
			Expert (E)	Organisational leader (OL)
Care homes	Social Care Working Group (SCWG 2020, 2021)	1	N/A	
Close contact retail	Canham et al. (2022)	2	N/A	
Construction	Balmforth et al. (2021), Bourne et al. (2022)	2	0	3
Energy production	Clabon et al. (2023)	Organisational contribution provided remotely (3 weeks after workshop)	0	2
Food and drink processing	Hosseini et al., (2022), Loh et al. (2022)	3	1	1
Higher education	N/A	2	1	0
Logistics/delivery	Wei et al. (2022), Whitfield et al. (2023)	2	0	1
Public transport	Coleman et al. (2022a, b)	2	1	2
Nonspecific	N/A	3	N/A	

N/A: not applicable.

**Fig. 1.**
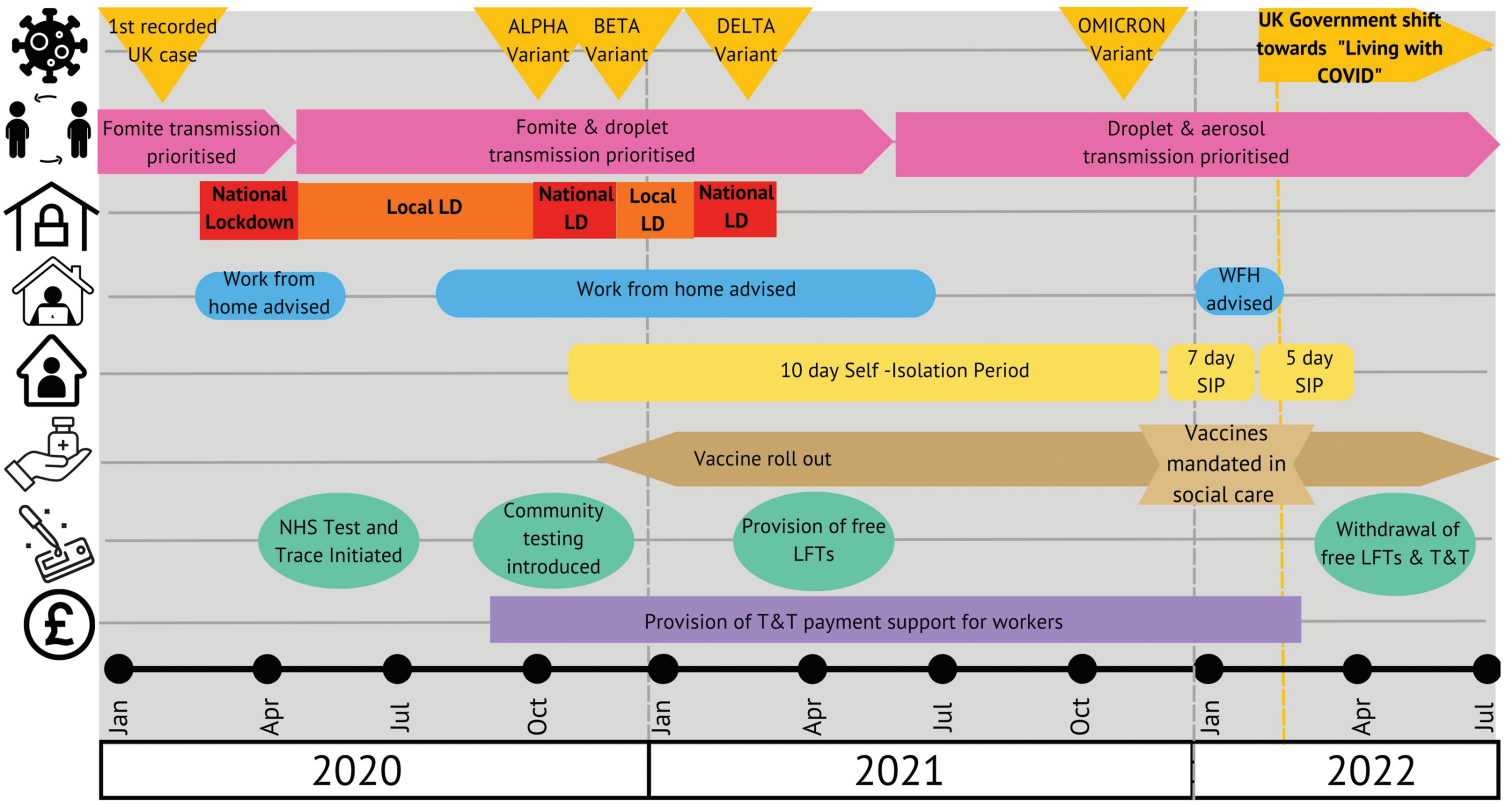
Timeline of COVID-19 Government policy and decision making in England.

As the COVID-19 pandemic developed, knowledge of how SARS-CoV-2 behaved in different circumstances evolved and guidance issued by the UK Government changed to remain in line with new knowledge. Figure 1 illustrates some of the changes in government knowledge (e.g. transmission was originally believed to be primarily via fomites, later by fomite and droplet but later on was understood to be airborne), regulation, and recommended practices (lockdowns, testing, and isolation) in England over the course of the COVID-19 pandemic. While initially similar in scope, the 4 UK nations soon amended their COVID-19 guidance and legislation in different ways and at different times (Paun et al. 2020). In February 2022, the UK Government announced a shift in their COVID-19 strategy from one of caution and prevention, to one of “living with COVID” (UK Government 2022), shifting the onus onto individual organisations to manage the ongoing risk of SARS-CoV-2 as part of their “business as usual”. The intentions being to manage COVID-19 like other respiratory illnesses, while minimising mortality, responding to potential new variants or waning immunity, protecting the National Health Service (NHS) from unsustainable pressures, and allowing the reduction of mitigations which were affecting “normal” working practices for so many industries/organisations. In general, the guidance was not sector specific and it was up to individual companies to make their own choices for removing/keeping mitigations.

LFT: lateral flow test, SIP: self-isolation period, and WFH: work from home.

Timeline constructed from: Alwan et al. 2020; BBC News 2021, 2022; British Foreign Policy Group 2022; Cabinet Office, 2021, 2022; Department of Health and Social Care 2020, 2021, 2022, 2023; House of Commons Library 2022; Institute for Government 2021; Nottinghamshire Live 2022; Office for National Statistics 2022; UK Health Security Agency 2020, 2022, 2023.

### Purpose of this research

To truly learn lessons for the future from the COVID-19 pandemic, it was important to look back at the sector-specific empirical research conducted between October 2020 and February 2022. This research sought to explore the extent of similarities and differences in sector-specific research findings. This is supplemented with fresh insight into risk perceptions, changes to practice, and pandemic recovery amongst the sectors of interest, to understand the changes in approach being taken in response to government guidance on “living with COVID” since February 2022 (e.g. mitigations retained, response for future SARS-COV-2 variants, and other health emergencies). Findings drawn from a variety of industry/organisational perspectives are collated to improve understanding of the perceived risks associated with SARS-COV-2 infection over time and support the specific sectors to return to more normal operation.

Ethical approval for primary data collection (Phase 2) was provided by the Reading Independent Ethics Committee (IOM P783 PROTECT NCS, 15/7/22).

## Methods

### Phase 1

We first investigated the similarities and differences in reported challenges to mitigating the risk of SARS-COV-2 transmission; barriers, and facilitators to responding effectively to COVID-19; implementation of mitigation measures; and gaps in knowledge from sector-specific research carried out as part of PROTECT National Core Study research between October 2020 and February 2022.

At the end of April 2022, a knowledge share workshop was conducted with PROTECT researchers who had led or contributed to the sector-specific research programme funded or affiliated with PROTECT. This included the following 8 sectors: care homes; close contact retail; construction; energy production (nuclear); food and drink processing; higher education; logistics/delivery; and public transport. A total of 14 PROTECT researchers participated, some of whom represented more than 1 sector or fulfilled a coordinating role across the PROTECT research strands not related to a particular sector (see Table 1).

During this in-person interactive workshop, participants were asked to complete 4 tasks to capture the knowledge gained from sector-specific research. These were to summarise reported:

1. COVID-19 mitigation measures put in place;
2. Challenges to mitigating the risk of SARS-COV-2 transmission;
3. Barriers and facilitators to preventing workplace transmission; and
4. Gaps in industry knowledge identified.

Participating researchers were only able to report on the findings that fell within the scope of their research (e.g. the close contact retail study focussed on face coverings only, hence researchers could not report on other mitigations, which may have also been present within this sector). Additionally, a concurrent PROTECT study was being undertaken within an energy production company (April 2022 onwards). Workshop contributions from this organisation was provided retrospectively in written form for integration into the analysis.

Workshop contributions were analysed thematically (Braun and Clarke 2006) within Excel, by 4 researchers with skills in qualitative research. Deductive analysis methods sought to identify important aspect of the data which helps to answer the research question. Inductive methods also sought to identify emergent patterns within the data corpus offering meaning of value to the topic under study. Following all data collection the relative prominence of findings were evaluated relating to the 8 sectors represented (Canham et al. 2022). As such, workshop findings were categorised in 1 of 3 ways:

- “Common findings”—identified across all or most of the sectors (i.e. 7 or 8 sectors);
- “Cross-cutting findings”—identified for between 3 and 6 sectors; and
- “Sector-specific findings”—identified for just 1 or 2 sectors.

### Phase 2

We subsequently, in Autumn 2022, sought to understand approaches implemented across the different sectors in response to government guidance on “living with COVID” issued in February 2022.

Up to 4 individuals from 6 of the sectors (construction; food, and drink processing; higher education; logistics/delivery; and public transport and an energy production company) were invited to participate in a semi-structured qualitative interview following their engagement in prior or concurrent sector-specific research (see Table 1). Eleven semi-structured qualitative interviews were conducted in total with 12 participants representing “organisational leaders” and “sector experts” (policy, academic).Interviews were not conducted with representatives of the close contact retail sector as Phase 1 research (Canham et al. 2022) concentrated on face covering usage only, or with care homes as prior research was not empirical in nature (rather modelling work and advice to Government) (Social Care Working Group 2020, 2021).

Interviews lasted between 30 and 60 min and were conducted during August and September 2022, by phone or using video conferencing technology (Zoom/Microsoft Teams). Interviews explored how sectors/organisations had responded to government guidance on the “living with COVID” phase of the pandemic. Topics addressed in these interviews were mitigations and managing COVID-19, worker sickness and support, knowledge gaps, learning lessons, and future challenges. These were informed by Phase 1 results and the evolving Government guidance for workplaces in the United Kingdom and were used to collect factual information on what had changed for sectors/companies in dealing with COVID-19 and trying to return to business as usual since February 2022. Written transcripts were again subject to qualitative thematic analysis (Braun and Clarke 2006) by the same 4 qualitative researchers during Phase 1 using Excel, to identify and code patterns of responses and reveal themes and trends in the data (Coleman et al. 2023). These were then compared and contrasted with findings emergent from Phase 1.

In both phases of the research, respondents voluntarily spoke about wider contextual issues such as BREXIT, the cost of living crisis, the war in Ukraine, and worker shortages, which were impacting on their decision making in addition to issues directly related to COVID-19. These are returned to in the discussion section of this article.

## Results

### Mitigation measures

Phase 1 revealed that all sectors introduced the following mitigation measures: improved ventilation; face coverings; social distancing; enhanced cleaning regimes; and COVID-19 testing. The type of testing (lateral flow testing [LFT], polymerase chain reaction testing [PCR], and antibody testing) varied by sector. Table 2 sets out mitigations mentioned by sector for both common (7–8 sectors) and cross-cutting (3–5 sectors) findings by researchers linked to specific sectors during the workshop.

**Table 2.** Mitigations by sector.

Mitigations	Sectors							
	Public transport	Food processing	Higher Education	Logistics/delivery	Close contact retail	Care homes	Construction	Energy
Common findings (identified in 7–8 sectors)								
Face coverings	√	√	√	√	√	√	√	√
Ventilation	√	√	√	√	√	√	√	√
Enhanced cleaning	√	√	√	√		√	√	√
Social distancing	√	√	√	√		√	√	√
Testing	√	√	√	√		√	√	√
Cross-cutting findings (identified in 3–6 sectors)								
Vaccination	√	√				√	√	√
Screens/physical barriers	√	√	√	√				√
Work bubbles	√	√		√				√
Work from home	√	√	√				√	√
Signage	√	√	√		√			
Reduced external contacts		√		√		√		√
Enforced isolation		√	√			√		
Transport restrictions	√			√				√
Shared facilities restrictions	√						√	√
Enhanced hand hygiene	√		√					√
PPE (gloves/aprons)	√			√		√		

A number (*n* = 22) of sector-specific (1–2 sectors) mitigation measures were also reported to have been introduced at different points during the pandemic. Examples of which for the different sectors included: public transport: shielding vulnerable staff; food processing: contact tracing; higher education: staff/student sickness management; logistics/delivery: exclusions zones; close contact retail: modification of practices (e.g. reduction in time spent in consulting rooms); care homes: use of antivirals; construction: preexisting safety culture; energy: site-based COVID hubs.

During initial sector-specific investigations, there was a general belief expressed from participants across most sectors, that the majority of mitigation measures would remain in situ for the foreseeable future, with planning ongoing by organisations to both encourage and police their usage. However, all participants interviewed during Phase 2 reported the majority of mitigation measures within their sector/organisation had been removed in response to the changes in Government guidance in February 2022. Furthermore, in the absence of freely available testing, COVID-19-related sickness absence was reportedly being captured under “business as usual” processes for monitoring staff sickness.

Participants in Phase 2 revealed that in some cases there were alternative reasons (beyond reduction of viral transmission risk) for maintaining mitigation measures within their organisation/sector. These included:

- continuation of enhanced cleaning on public transport, maintained for reasons of public confidence to encourage the return of passenger footfall (referred to as “hygiene theatre” amongst sector representatives) and
- use of thermal imaging cameras within energy production, intended as a visual deterrent for workers attending site if feeling unwell.

Vaccination, as a mitigation measure presented a common thread for reflection throughout both phases of this research. Vaccination was recorded as a cross-cutting mitigation measure amongst 5 of 8 sectors (care homes, construction, energy production, food and drink processing, and public transport) for Phase 1. Variations in uptake of vaccinations amongst workers was also recorded as a transmission risk across 7 sectors (all but energy production). Only the participant(s) from the energy company reported that they continued collecting vaccination data after February 2022, although information provision was voluntary for workers. The following quotes illustrate these points:

We’ve obviously got a lot of the influenza strains covered by flu vaccine, but not that many of our staff and students will be eligible [see Note 1] for flu vac [sic] and the COVID booster as well. (Higher Education Expert)

We also monitor people’s vaccination details, so we’ve got a way of recording people’s first, second, and third doses. (Energy Production Organisational Leader)

The energy production, logistics/delivery, and public transport sectors reported delayed removal of some mitigations after February 2022, wanting to increase worker/public confidence, and giving the companies time to monitor data on COVID-19 case rates in the wider community. The energy company appeared to have retained more mitigation measures for longer than other sectors in this study, including continued provision of free test kits to staff. This is perhaps unsurprising given that this company contributes to the UK’s critical national infrastructure and employs a highly specialised workforce.

### Challenges to mitigating the risk of SARS-COV-2 transmission

During Phase 1, 6 challenges to mitigating risk of SARS-COV-2 transmission were identified across all 8 sectors. These included compliance fatigue; attitudes to testing and isolation; changing/differing guidance; lack of sick pay/financial support; and living in shared accommodation and use of shared transportation. Workers and members of the public reportedly experienced compliance fatigue. Lack of financial support to enable self-isolation or sickness absence away from the workplace related to that provided by the government and/or employers. Lack of financial support effectively forced workers to make the choice between getting paid and risking making friends/colleagues sick. Differences between contract terms (e.g. gig workers, contractors, and agency staff when compared to direct employees) and company policies were also believed to increase the potential for inequality amongst different worker groups with regard financial support.

During Phase 2, compliance fatigue was again cited (construction, logistics/delivery, and public transport) as a barrier to controlling viral transmission. Indeed, workers were reportedly less willing to undertake protective practices (e.g. wearing face coverings and social distancing) in work environments after guidance changed for society in February 2022. This is illustrated by the following quote:

It’s not at the forefront of people’s minds anymore, so it makes it very difficult behaviourally, to get people to follow certain rules […] I think the perception is probably the biggest challenge now. (Construction Organisational Leader)

During Phase 2, respondents from construction, energy production, and higher education made reference to encouraging their workers/service users to be “good citizens” (e.g. encouraging them to remain at home in the event of illness and reporting respiratory tract infections to avoid spreading illness at work). Participants recognised that this was harder for some, dependent on their contract terms and company policies. Some organisational leaders had however made changes to help address such inequalities, as illustrated by the following quote:

…somebody like me, I get full sick pay for a year, the operatives are straight onto statutory sick pay. We’ve had a change in terms of our policies, whereby everybody gets the same sickness absence and flexible working for as many people as possible. (Construction Organisational Leader)

### Enablers and barriers to preventing the spread of the virus

During Phase 1, the availability and use of testing was the only commonly cited enabler to preventing viral transmission identified across 7 sectors (not close contact retail). By Phase 2, many participants perceived the rates of SARS-COV-2 to be low, but described feeling as though they had lost “early warning signs” for increasing case rates, as active testing data collection had stopped. This was due to cessation of NHS contact tracing (i.e. “test and trace”), removal of freely accessible COVID-19 testing, and associated sickness absence monitoring, now captured as “business as usual.” The following quote illustrates this perspective.

We seriously monitored…I mean, to within an inch of our lives monitored COVID and the implications of COVID for our business. As things started to tail off and things became business as usual it became part of the landscape of normal sickness absence. (Logistics/delivery Organisational Leader)

During Phase 2, participants were keen to highlight the benefits of partnership/collaborative working, to share best practice, learn lessons, and keep sectors operational. Some industry partnerships/collaborative forums were said to be continuing at the time of interview (e.g. higher education, public transport). This contrasted with Phase 1, where poor collaboration/partnership working was highlighted as a common challenge to risk mitigation of viral transmission amongst 7 of the 8 sectors (not energy production).

Two common barriers cited by almost all sectors during Phase 1 related to communication of messaging and confusing government guidelines. Organisations were said to have been confused and frustrated by the frequency at which they were required to update their communications and mitigation measures in response to changing government guidelines, often at very short notice. In addition, the cost, to both employers (e.g. installing mechanical ventilation, setting up site-based testing) and employees (e.g. loss of income through sickness absence/isolation or travel costs due to restrictions on vehicle sharing), was noted as a common barrier across all sectors.

If I look across government and [other bodies], everybody was publishing kind of their own set of guidance, and I do think what’s really important is you have a single authoritative voice saying, this is what we’re going to do, these are the rules and this is why they’re in place and this is what we expect you to do and everything feeds from that. (Public Transport Organisational Leader)

During Phase 2, participants acknowledged that powers of enforcement related to COVID-19 had been significantly reduced. Interview participants (construction, higher education, logistics/delivery, and public transport) perceived this to have made it more challenging for them to formally apply/enforce mitigations and access accurate data on which to base their ongoing decision making. The following quote illustrates this.

… because of the paucity of data, we’ve known that cases went up, but it’s been hard to pinpoint any additional prevention and control measures that were needed, and testing and—sorry, Test and Trace stopped as well. (Higher Education Expert)

Once Government messaging changed under the “living with COVID” regulations, sectors and organisations had limited levers via which to force compliance with COVID mitigations from their workforce. Some companies (energy production, delivery and logistics, and public transport) chose to slightly delay lifting some or all mitigations while examining the impact of doing so on SARS-COV-2 infection rates in the wider community. However, this was only partially successful due to compliance fatigue and confusion over rules being different within and outside the workplace. Government messages ideally need to be in place, clear, consistent, supported, and policed to make this happen. However, some organisations may be better able to justify more stringent rules to their workforce, due to their inability to shut down as part of the UKs national infrastructure (e.g. energy production).

### Gaps in COVID-19 knowledge

During Phase 1, a number of cross-cutting knowledge gaps were identified across multiple sectors. Many of these related to mitigation measures, such as understanding the effectiveness of individual measures and which measures to continue implementing in preparation for future variants of SARS-CoV-2. In many sectors/organisations, multiple mitigation measures had been introduced simultaneously or in quick succession and hence their relative impact and cost effectiveness was not known. Other knowledge gaps identified related to 3 key topic areas:

- Environmental conditions (e.g. ventilation, humidity, and temperature), with concern for the relative impact on transmission risk within the workplace.
- Behaviour and communication, specifically related to how best to plan and communicate risk-related information and behavioural requirements to different groups, and indeed insight into “at risk” worker populations within their respective sector/organisation.
- Long-term issues, such as likely transmission routes, changing symptoms of the SARS-COV-2 virus, and how best to plan for the future (e.g. uncertainty over the medium and longer term impacts of the governments “living with COVID” guidance and emergency preparedness for future health challenges).

During Phase 2, some of the aforementioned knowledge gaps remained prominent with many participants still unclear about environmental conditions and associated challenges to mitigating the risk of SARS-COV-2, as illustrated by the following quotes:

The ventilation systems are very complex and different […] So you won’t see a pattern because there are so many opportunities for transmission to occur besides the X number of hours, the person has to be standing at a workstation. […] I think there was probably a knowledge gap of understanding what good quality air is (Food and Drink Processing Organisational Leader)

Knowing more about the transmission, the rate of transmission and the areas of risk would be helpful […] What are the most effective controls for our work environment? (Construction Organisational Leader)

A visible shift in preparedness could however be seen amongst participants during Phase 2, most of whom felt better prepared to respond in the event of a future health emergency. A lack of available data through which to monitor rates of COVID-19, however, gave cause for concern, particularly within the higher education sector, over the ability to detect rising rates or new variants which would enable subsequent action to be taken.

Participants in the construction, logistics/delivery, and public transport sectors also highlighted challenges with differing government guidance being issued across the 4 UK nations due to devolution where some powers have been passed from Westminster (England) to elected bodies in Cardiff (Wales), Belfast (N Ireland), and Edinburgh (Scotland) since 1997/1978. This was said to make it challenging to maintain operations and issue appropriate corporate communications amongst businesses that cut across 2 or more UK countries (e.g. train operators running services travelling through multiple UK countries).

### The wider context

During Phases 1 and 2, participants referred to challenges beyond COVID-19 threatening the operation of their business/sectors. This included Great Britain’s exit from the European Union (BREXIT) (Phases 1 and 2), the cost of living crisis, and the war in Ukraine (Phase 2 only). Such external influences were said to be driving up the costs of products and services, contributing to worker shortages, demanding redirection of attention, and therefore diminishing time and resource to prepare for future emergencies. Such points are illustrated by the following quotes:

So, I know from a resource point of view a number of them [contract workers] have gone and started to drive for [named delivery company] because the wages are, kind of, good and they get to go home. So, we’ve had a few individuals from a supply base have, kind of, changed careers. (Energy Production Organisational Leader)

Most industries right now are struggling for people, across the board, and that is driving up wages and competition. (Public Transport Organisational Leader)

## Discussion

The detailed findings from both phases of the study make clear that different sectors, and indeed sub-sectors, have differing workplace characteristics, practices, and workforce demographics which need consideration when enabling sectors to recover from the pandemic and planning for any future public health crisis. For example,

- Some sectors (construction and logistics/delivery) generally had poorer employment contracts (e.g. zero hours) and well-being benefits (e.g. sick pay) and, due to the nature of the work, were more likely to use shared transportation.
- As part of the nation’s critical infrastructure, the energy production company had to find a balance between maintaining operation and maintaining worker safety.
- The higher education sector has a younger, more mobile population (students) who have close interactions with workers.
- The food and drink processing sector has unique and varied operational activities and environments (e.g. working outdoors picking produce is very different to a close proximity indoor production line) as well as large variation in workforce characteristics (e.g. increased proportions of migrant workers in some sub-sectors).
- The need to overcome government messaging, which advised against travel on Public transport at the height of the pandemic, to increase ridership and improve revenue is important as Government subsidies to the sector have ended.

The challenges of controlling SARS-COV-2 transmission are therefore not homogenous and hence mitigation measures, barriers, and enablers are likely to be different across the multiple sectors and settings. It is therefore imperative that organisations/sectors are enabled to tailor their control strategies to maximise their effectiveness.

Participants from the energy production, higher education, logistics/delivery, and public transport sectors also discussed measures to align risk management approaches and/or communications—between business sites, within the sector, across sectors, and/or across countries, as illustrated by the following quote.

… this is a dispersed delivery model that we operate. How do we ensure that communications are consistent […] science and evidence are major competing narratives here. So I think a key lesson is about getting facts straight and communicating those back to the sector. (Public Transport Expert)

While many of the organisational leaders had shifted their focus to recovery in the short term and getting back to “business as usual,” during Phase 2, the experts interviewed were still reporting spending a significant amount of their time developing contingencies for any potential COVID-19 increase. This generally related to concerns over: the emergence of potential new COVID-19 variants and seasonal differences (autumn/winter); reductions in vaccination uptake (no longer offered to under-50 s along with increasing apathy around boosters (Ryan 2022)); and longer term policy and strategy to combat potential health emergencies, including COVID-19 (e.g. ventilation systems/air quality monitoring, worker policies related to sick pay, and mental health support).

Throughout both phases of this PROTECT research study, the majority of participants expressed difficulty in responding to the changing rules and regulations issued by UK Government. Organisations with nationwide operations also struggled to bridge the gap between differing guidance across 4 UK nations in some sectors (construction, logistics/delivery, and public transport). The burden and cost for sectors responding to changing guidance was commonly acknowledged as a challenge, heightened by external factors (e.g. cost of living crisis, Ukraine war, etc.), unrelated to COVID-19, demanding attention and resource. Furthermore, a lack of understanding amongst workers and the public of why the rules, and changes to these rules over time, were necessary often compounded these issues. In this regard compliance fatigue, the reduction of enforcement powers and loss of data sources through which to actively monitor COVID-19 were said, during Phase 2, to make it more challenging for organisations to apply and enforce mitigation measures during the “living with COVID” phase.

Another common thread to emerge from both Phases of the research was a wish to understand which mitigations worked best, in what contexts and their relative cost effectiveness. During the “living with COVID” phase, experts and organisational leaders remained unsure about which measures were the most impactful for their organisations, workers, and service users. This was due to the introduction of multiple measures simultaneously, likely compounded by the diverse challenges to mitigating the risk of SARS-COV-2 transmission evident in different sectors, sub-sectors, organisations, or even workplace settings. Indeed, layering control strategies in any given context was cited by some as an effective strategic approach (Canham et al. 2023).

Participant’s views and organisational practices changed quickly during the different phases of the pandemic. Following removal of the majority of restrictions, most sectors/organisations were keen to restore normal practice, thereby treating COVID-19 like any other illness, while planning for future stability and preparing for future health emergencies.

It is essential that those making policy at the national level and decision makers in organisations are mindful of the lessons learned and knowledge gained at all levels (national, regional, local, organisational, and individual) during the COVID-19 pandemic. Therefore, recommendations are proposed for: managing ongoing recovery, as sectors/organisations continue “living with COVID” and other respiratory diseases (e.g. seasonal flu); balanced with longer-term planning for the next public health crisis (discussed below).

## Conclusion and recommendations

**Fig. 2.**
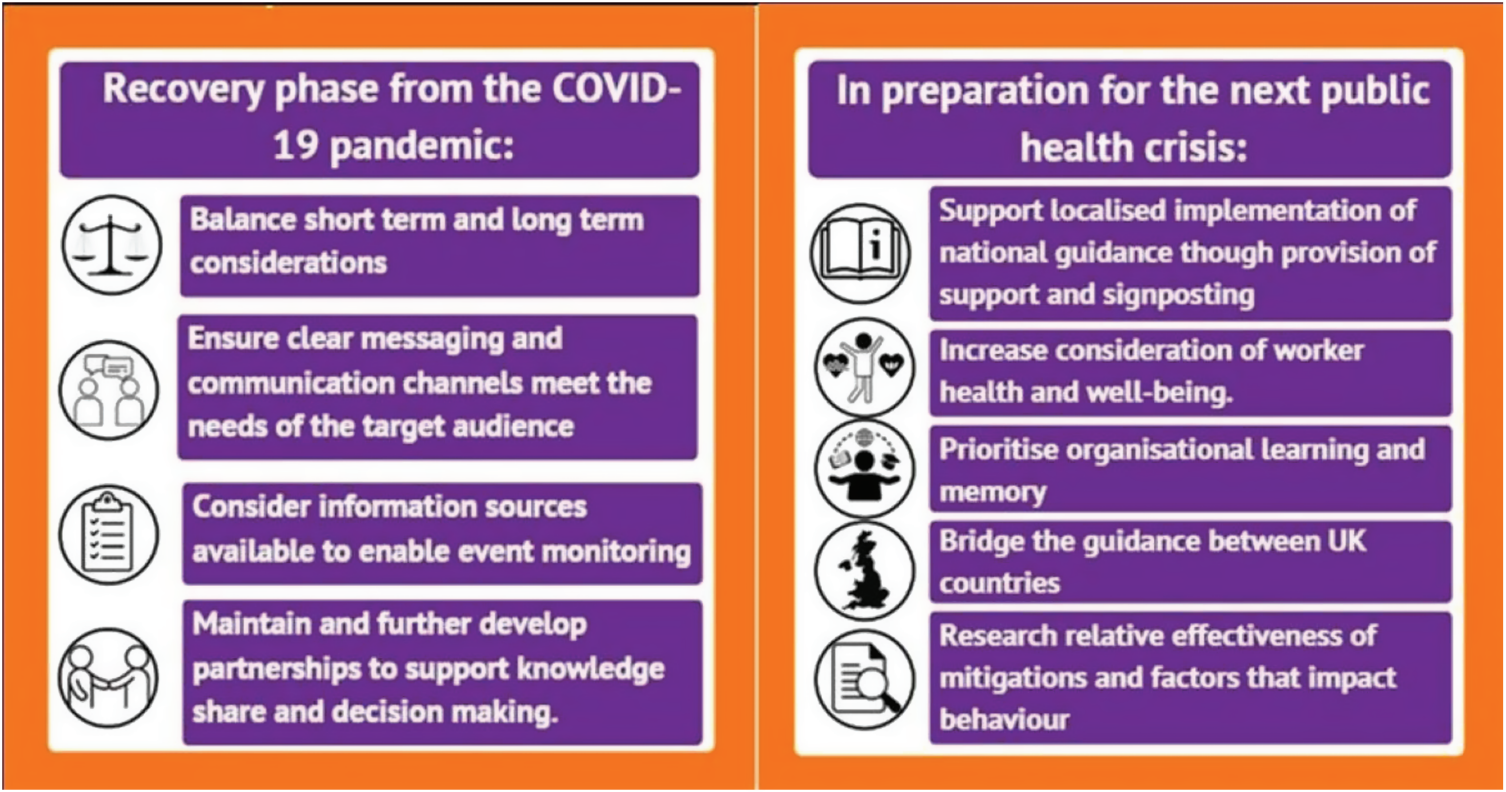
Recommendations.

A number of recommendations informed by the 2 phases of our research are proposed to support sectors and organisations recovering from the COVID-19 pandemic and planning for the next public health crisis, as illustrated within Fig. 2.

### Recovery from the COVID-19 pandemic

Overarching factors with immediate implications for sector/organisational operation, such as BREXIT, the cost of living crisis, and worker shortages, need to be considered alongside potential increased rates of COVID-19 seasonally and the introduction of eligibility restrictions for accessing COVID-19 boosters (NHS Website 2023). Longer term planning for business continuity should consider actions with long-lasting impact, for example, the design of buildings, improvements to ventilation (in buildings or on vehicles), etc. The development of effective, clear, and accurate messages, communicated well through appropriate channels are essential to keeping workers and the public engaging with services as safely as possible. It is easy to see how the key message and its reasoning could be lost during the required cascading and interpretation of messaging from Government Departments down through sectors, organisations, and ultimately to individual workers and the public. The maintenance of noninvasive event monitoring within businesses and across sectors should be given careful thought, and policies should be implemented, and information provided that help aid workers “do the right thing” when they become unwell (e.g. sick pay, well-being services). Additionally, sectors should be encouraged to maintain the partnerships established during the pandemic (e.g. food and drink processing and public transport), which have helped with joint decision making within and across sectors for relevant matters beyond COVID-19. These will enhance knowledge and practice sharing and may contribute to the provision of consistent messages for workers and the wider public.

### Preparation for the next public health crisis

The importance of national guidance is clear; however, advice and policy guidance must also support and enable localised implementation at the sector, subsector, and organisational level if it is to address the challenges to mitigating the risk of SARS-COV-2 transmission specific to the workforce and characteristics of the work environment (Hartwig et al. 2022). The focus on profitability and business operations displayed by some organisations may cause or contribute to worker ill health as well as risk damaging the reputation of the organisation. Greater consideration should therefore be afforded to protecting the well-being of workers through further development of organisational policy (e.g. improvements to the provision of sick pay, not currently in place for some workers). Organisations should take care to maintain organisational memory related to COVID-19 by reviewing policies, reflecting on practices, and updating contingencies periodically to ensure knowledge is retained and prompt action achievable when required. Different rules and requirements in place across the 4 UK nations caused frustration and confusion for organisations and their workers with operations that crossed UK country borders. Therefore, consideration should be given to bridge the guidance between these countries and maintain consistency where possible.

Further research into mitigation measures is needed to increase knowledge regarding their relative effectiveness in reducing COVID-19 transmission and cost effectiveness in different settings, and would be beneficial, in order for sectors to better prepare for future health crises. Additional research exploring the behavioural factors that affect compliance (Armitage et al. 2023), and how this may change over time, is also recommended to help develop future policy and practice.

## Note

^1^Due many students and staff being under the age of 50 and not eligible for free flu jabs or COVID boosters at the time of interviews.

## Data Availability

Data available for Phase 1 (workshop) can be found and reproduced by Canham et al. (2023). The data underlying this article for Phase 2 (interviews) cannot be shared publicly due to it being generated via qualitative interviews where participants were guaranteed anonymity.
